# Evaluation of thrombolytic potential of three medicinal plants available in Bangladesh, as a potent source of thrombolytic compounds

**Published:** 2014

**Authors:** Ali Ramjan, Marjan Hossain, Jannatul Ferdous Runa, Hasanuzzaman Md, Islam Mahmodul

**Affiliations:** 1 *Department of Pharmacy, Noakhali Science and Technology University, Sonapur, Noakhali- 3814, Bangladesh*

**Keywords:** *In vitro*, *Streptokinase thrombolytic activity*, %* Clot lysis*

## Abstract

**Objective: **The present study is aimed to investigate *in vitro* thrombolytic activity of three Bangladeshi medicinal plants *Averrhoa bilimbi* (Oxalidiaceae), *Clerodendrum viscosum* (Verbanaceae) and *Drynaria quercifolia* (Polypodiaceae).

**Materials and methods: **Each the plant was extracted with methanol at room temperature and the concentrated methanolic extracts (MEF) were fractionated by the modified Kupchan partitioning method to render pet-ether soluble fraction (PESF), carbon tetrachloride soluble fraction (CTSF), chloroform soluble fraction (CSF) and aqueous soluble fraction (AQSF). To observe their thrombolytic potential, a prompt and swift method was involved where streptokinase and water were used as positive and negative control, respectively.

**Result: **Among the three plants, AQSF and PESF of *D. quercifolia *with CTSF of *C. viscosum *exhibited highest thrombolytic activity by clot lysis of 34.38%, 34.27% and 28.64%, respectively. Among other extracts *A. bilimbi*, *C. viscosun* and *D.quercifolia *showed significant percentage (%) of clot lysis compared to standard streptokinase (41.05%) while the negative control water revealed 3.31 % lysis of clot.

**Conclusion: **From our findings it is observed that all the plants revealed remarkable thrombolytic activity. Therefore, steps should be taken to observe *in vivo* clot dissolving potential and to isolate active component(s) of these extracts.

## Introduction

Recently blood clot formation has been a severe problem of blood circulation. Thrombus or embolus hinders the blood flow by blocking the blood vessel therefore depriving tissues of normal blood flow and oxygen. These consequence yield necrosis of the tissue in that area. Thrombin formed blood clot from fibrinogen and is lysed by plasmin, which is activated from plasminogen by tissue plasminogen activator (tPA). The purpose of a fibrinolytic drug is to dissolve thrombin in acutely occluded coronary arteries thereby to restore blood supply to ischemic myocardium, to limit necrosis and to improve prognosis (Laurence et al., 1992 ▶).

For the treatment of myocardial infarction, many thrombolytic agents are used. Among them, streptokinase is remarkable and widely used. Moreover, Tissue-type Plasminogen activator is more effective and safer than either urokinase or streptokinase type activators. It is noted that all available thrombolytic agents still have significant deficiencies, including the necessity of large doses to be maximally effective, limited fibrin specificity and a significant associated bleeding tendency. Therefore, steps are taken to develop improved recombinant variants of these drugs in order to minimize deficiencies of the available thrombolytic drugs (Adams et al., 1991 ▶; Nicolini et al., 1992 ▶; Lijnen et al., 1991 ▶; Marder 1993 ▶; Wu et al., 2006 ▶).

*Averrhoa bilimbi *Linn. belongs to the family of Oxalidaceae having some local name including belembu, belemburi; In English, this is also familiar with- bilimbi, cucumber tree, tree sorrel. The plant has a short trunk soon dividing into a number of upright branches; attractive, long-lived tropical tree, reaches 16 to 33 ft. (5-10 m) in height. Probably, *A. bilimbi *is inborn of the Moluccas in Indonesia. This plant is also available in Brazil, Cuba, Philippines, Sri Lanka, Bangladesh, Myanmar (Burma) and Malaysia (Hasanuzzaman et al., 2013 ▶). *A. bilimbi *is medicinally used as a folk therapy for many purposes including antibacterial, antiscorbutic, astringent; postpartum protective medicine. It is also used for the management of fever, mumps, pimples, inflammation of the rectum and diabetes, itches, boils, rheumatism, syphilis, bilious colic, whooping cough, hypertension, stomach ache, aphthous ulcer and as a cooling drink (Roy et al., 2011 ▶).

*Clerodendrum viscosum* Linn. belongs to the family of Verbanaceae, commonly known as Bhat in Hindi and Ghentu, in Bengali, is a terrestrial shrub having square, blackish stem and simple, opposite, decussate, petiolate, exstipulate, coriacious, hairy leaves with a disagreeable odor (Kirtikar et al., 2001 ▶). The shrub is about 2-4 feet in height. Various parts of the plant are used by tribes for the treatment of colic, scorpion sting and snake bite, tumors and certain skin diseases, while the leaves are somewhat bitter, cure inflammation, skin diseases and good in small pox (Santanuet al., 2009 ▶).

*Drynaria quercifolia* J. Smith belongs to the family of Polypodiaceae, locally known as Gurar, is a parasitic fern (Bhattacharya 1990 ▶; Kirtikar et al., 1994 ▶) that is generally distributed in Bangladesh, India and Thailand. The rhizomes of the plant have antibacterial properties and are used traditionally for management of cough, tuberculosis and typhoid fever. ASEAN Centre for Biodiversity stated in their Checklist of Medicinal Plant in Southeast Asia that rhizome decoction or drink of *D. quercifolia* rhizome uses as antipyretic preparation (Ramesh et al., 2001 ▶).

## Materials and Methods

**Collection and identification**

Different parts of *Averrho abilimbi* (fruits), *Clerodendrum viscosum* (leaves) and *Drynaria quercifolia *(leaves) were collected from Noakhali, Bangladesh during the month of July 2013 and voucher specimens for each of the collections (DACB 37752, 35979 and 37654 respectively) have been deposited in Bangladesh National Herbarium (BNH) for future references. Different parts of the plants were washed with clean water to discard dirt materials, dried under shade with casual sun drying, ground into coarse powder and preserved in a closed container at 25°C for further use.

**Preparation, extraction and fractionation of plant material**

The extraction was performed using cold maceration technique. Powder portions of different plants (500 g) were soaked in 2500 ml of methanol for about 10 days at room temperature with occasional stirring. The solution was filtered through filter cloth followed by Whatman’s filter paper and the filtrate thus obtained was concentrated designated as MEF. This was done by evaporation method under ceiling fan and in a water bath below 40°C temperature. The concentrated MEF was partially separately by modified Kupchan method (Vanwagenen et al., 1994 ▶) using chloroform, carbon tetrachloride and pet-ether to yield CSF, CTSF and PESF. Aqueous portion was retained as AQSF. Four organic solvent soluble fractions were dried for 7 days at room temperature by evaporation method.

**Streptokinase (SK)**

Streptokinase (15, 00,000 I.U.,) used as a standard which was collected from Beacon pharmaceutical Ltd, Bangladesh. 5 ml sterile distilled water was added to streptokinase vial and mixed properly. From this suspension100μl (30,000 I.U) was used for *in vitro* thrombolysis (Prasad et al., 2007 ▶).

**Preparation of sample**

The thrombolytic activities of all plant extracts were evaluated by a method using streptokinase (SK) as a reference standard. 100 mg of MEF, CSF, CTSF, PTSF, and AQSF of different plants were dissolved respectively in 10 ml of methanol, chloroform, carbon tetrachloride, pet-ether, distilled water and were kept overnight. Then the soluble supernatant was decanted and filtered.

**Blood sample**

Blood samples were collected from healthy human volunteers (n=5) by maintaining aseptic condition without a history of oral contraceptive or anticoagulant therapy. 1ml of blood was transferred to the previously weighed microcentrifuge tubes to form clots. Study protocol was approved by ethical committee of Pharmacy department, Noakhali Science and Technology University, Noakhali, Bangladesh. Written consent was obtained from each volunteer prior to collect blood sample.

**Thrombolytic activity**

5 ml of venous blood were drawn from each volunteers which were taken in five different pre weighed sterile micro centrifuge tube and allowed to incubate at 37 °C for 45 minutes. After clot formation, fluid was completely released from each microcentrifuge tubes and determined clot weight by subtracting weight of clot containing tube from weight of tube alone. As a positive control, 100 μl of streptokinase (SK) and as a negative non thrombolytic control, 100 μl of distilled water along with 100 μl of each samples were separately added to the microcentrifuge tubes. All the tubes were then incubated at 37 °C for 90 minutes and observed for clot lysis. After incubation, the released fluid was discarded and tubes were again weighed to observe the difference in weight after clot disruption. Finally percentage of clot lysis was determined as followings:

% of clot lysis = (wt of released clot /clot wt) × 100

**Statistical analysis**

Data obtained were analyzed using SPSS version 16.0 (SPSS Inc. Chicago, IL. USA). All values are expressed as mean±SEM for three replicates. Data were analyzed by one-way ANOVA and the statistical significance differences were analyzed using paired t- test. p<0.05 was considered statistically significant.

## Results

The thrombolytic activity of *A.bilimbi, C. viscosum *and* D. quercifolia *extracts were determined as a part of exploration of cardio protective drugs from plants reserves, and the conclusion are conferred in Table 1. It presents 41.05 % lysis of clot in a process of subsequent incubation for 90 minutes at 37°C, where 100μl SK was added as a positive control (30,000 I.U.). On the other side, it showed negligible percentages of lysis of clot (3.31 %), when we use distilled water as a negative control.

In this observation, highest thrombolytic activity (34.38%) is demonstrated by the AQSF of *D. quercifolia. *Besides, convincing thrombolytic activity were also exibited by PESF of *D. quercifolia *(34.27%), CTSF of *C. viscosum *(28.64%) and *A. bilimbi* (27.72%), PESF of *A. bilimbi* (27.5%) and *C. viscosum* (25.29%), AQSF of *C. viscosum* (24.39%), MEF and CSF fraction of *A. bilimbi* (23.94% and 23.51%).

Besides, MEF and CTSF of *D. quercifolia, *exhibit 23.46% and 20.33% of clot lysis respectively while AQSF of *A. bilimbi* exhibit 17.06% of clot lysis. On the other hand, MEF and CSF of *C. viscosum *along with CSF of *D. quercifolia *showed negligible amount of clot lysis. Figure 1 represents comparative thrombolytic activity of *A. bilimbi, C. viscosum* and *D. quercifolia *for each extract.

**Table:** Thrombolytic activity (in terms of % of clot lysis) of *A. bilimbi, C. viscosum* and *D. quercifolia*

**Treatment**	**% of clot lysis**		
	*A.bilimbi*	*C.viscosum*	*D.quercifolia*
**MEF**	23.94 ± 7.58**	11.25± 2.32***	23.46± 5.53**
**PESF**	27.5 ± 6.89**	25.29± 6.80**	34.27± 1.34*
**CSF**	23.51 ± 4.92**	4.1± 6.76***	6.16± 7.45***
**CTSF**	27.72 ± 4.92**	28.64± 3.76*	20.33± 4.81**
**AQSF**	17.06± 6.99**	24.39± 4.98**	34.38± 5.33*

Results represented in means ± SD (n = 3); Level of Significance

*** p<0.001,

** p<0.01,

* p<0.05 comparing with standard Streptokinase (41.05%). Here, MEF-methanolic extract fraction, PESF- pet-ether soluble fraction, CSF-chloroform soluble fraction, CTSF- carbon tetrachloride soluble fraction, AQSF-aqueous soluble fraction

**Figure:**
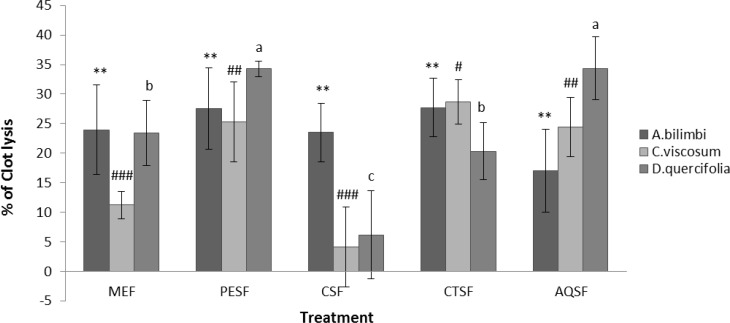
Comparative thrombolytic activity of *A. bilimbi, C. viscosum* and *D. quercifolia *for each extract. Significant at ^**^p<0.01 and for *A. bilimbi*, ^###^p<0.001, ^##^p<0.01 and ^#^p<0.05 for C. viscosum, and ^a^p<0.001, ^b^p<0.01 and ^c^p<0.05 for D. quercifolia when compared to standard streptokinase. Here, MEF-methanolic extract fraction, PESF- pet-ether soluble fraction, CSF-chloroform soluble fraction, CTSF- carbon tetrachloride soluble fraction, AQSF-aqueous soluble fraction

## Discussion

From the beginning of civilization, human are dependable on plants for the treatment of many diseases, Nowadays phytopharmacological investigation has created a new field to discovery plant derivative drugs, which are effective in remedial of certain diseases, and renewed the attention in herbal medicines. It is estimated that about 30% of the pharmaceuticals are prepared from plants derivatives (Leta et al., 2002 ▶; Gillman et al., 1995 ▶). A number of research works have been conducted to discover the plants and natural food sources and their supplements having antithrombotic (anticoagulant and antiplatelet) effect and there is indication that consuming such food leads to prevention of coronary events and stroke (Ratnasooriyaet al., 2008 ▶; Joshipura et al., 1999 ▶; Liu et al., 2000 ▶; Bazzano et al., 2002 ▶). Although there are several thrombolytic drugs with those obtained by recombinant DNA technology, but side effects related to some of these drugs that lead to further difficulties have been reported (Baruah et al., 2006 ▶; Gallus et al., 1998 ▶; Wardlaw et al,. 2004 ▶; Capstick et al,. 2005 ▶).

 Platelets play a significant role in the development of atherothrombosis as well as damage the regions of endothelial surface (produced by reactive oxygen species). The stimulated platelets form platelets to platelets bonds, binds also to leucocytes carrying them into an intricate process of plaque development and progression (Prenticeet al., 1999 ▶). Plasmin, a natural fibrinolytic agent, lyses clot by breaking down the fibrinogen and fibrin contained in a clot. Streptokinase forms a 1:1 stoichiometric complex with plasminogen that can convert additional plasminogen to plasmin (Banerjee et al., 2004 ▶). Moreover, phlorotannin, isolated from marine brown algae, have a unique property in promotion of dissolution of intravascular blood clot via antiplasmin inhibition (Prasad et al., 2007 ▶). Several studies reveal that *A. bilimbi, C. viscosum* and *D. quercifolia* possesses tannin, alkaloid saponin (Hasanuzzaman et al., 2013 ▶; Runa et al., 2013 ▶; De et al., 2013 ▶) which could be participated for its clot lysis activity (Ali et al., 2013 ▶)

The present study was carried out to investigate thrombolytic activity of three plants available in Bangladesh. Streptokinase (SK), a known thrombolytic drug is used as a positive control (Prasad et al., 2007 ▶). Water, on the other hand, was designated as a negative control. The comparison of positive control with negative control clearly confirmed that clot dissolution does not occur when water was added to the clot. By comparing with this positive & negative control, a significant thrombolytic activity was observed after treating the clots with *A.bilimbi*, *C. viscosum *& *D. quercifolia *extracts.

In conclusion from our recorded data, it can be demonstrated that our findings may have significant implications in cardiovascular health. In addition, this finding may indicate the possibility of developing novel thrombolytic compounds from *A. bilimbi, C. viscosum, *and *D. quercifolia *extracts. Further studies are ongoing to isolate and characterize the compounds responsible for thrombolytic activity
